# Network Pharmacology and Molecular Docking Analysis Exploring the Mechanism of *Tripterygium wilfordii* in the Treatment of Oral Lichen Planus

**DOI:** 10.3390/medicina59081448

**Published:** 2023-08-10

**Authors:** Wenkai Huang, Xu Huang, Lin Yang, Wenjia Han, Zhongqing Zhu, Yuanyin Wang, Ran Chen

**Affiliations:** College & Hospital of Stomatology, Key Lab. of Oral Diseases Research of Anhui Province, Anhui Medical University, No. 81, Meishan Road, Shushan District, Hefei 230032, China; hwk20220816@163.com (W.H.); hx2245010658@163.com (X.H.); yl997013672@163.com (L.Y.); 18055792108@163.com (W.H.); zzq13966082513@163.com (Z.Z.)

**Keywords:** *Tripterygium wilfordii*, oral lichen planus, network pharmacology, molecular docking

## Abstract

*Background*: Oral lichen planus (OLP) is an infrequent autoimmune disease of the oral mucosa, which affects up to 2% of the world population. An investigation of *Tripterygium wilfordii*’s mechanism of action for treating OLP was conducted, and a theoretical basis was provided for improving current treatment regimens. *Materials and Methods*: We used a network pharmacological approach to gain insight into the molecular mechanism of *Tripterygium wilfordii* in the treatment of OLP. Then, potential protein targets between *Tripterygium wilfordii* and OLP were analyzed through a drug–target network. This was followed by KEGG enrichment analysis and Gene Ontology (GO) classification. Finally, for molecular docking, AutoDock Vina was used. *Results*: A protein–protein interaction (PPI) network was constructed by analyzing the common targets of a total of 51 wilfordii–OLP interactions from different databases. The GO and KEGG enrichment analyses showed that the treatment of OLP with *Tripterygium wilfordii* mainly involves lipopolysaccharide response, bacterial molecular response, positive regulation of cytokine production, and leukocyte proliferation, and the signaling pathways mainly include the AGE-RAGE, NF-κB, Toll-like receptor, IL-17, HIF-1, and TNF signaling pathways. The molecular docking results showed that β-sitosterol, kaempferol, hederagenin, and triptolide have a higher affinity for AKT1, TNF, CASP3, and PTGS2, respectively. Based on the CytoNCA analysis of common targets, 19 key targets, including AKT1, TNF, VEGFA, STAT3, CXCL8, PTGS2, TP53, and CASP3, and their connections were identified. *Conclusions*: Preliminarily, this study reveals that *Tripterygium wilfordii* interferes with OLP by interacting with multiple targets through multiple accesses, as validated by molecular docking.

## 1. Introduction

Oral lichen planus (OLP), an inflammatory autoimmune disease of the oral mucosa, is characterized by chronic or recurrent disease. According to the WHO guidelines, OLP is designated as an oral pathology that may lead to malignancy, and one of its most dangerous complications is the development of oral squamous cells [1]. Currently, there is no effective cure for this disease. A commonly used treatment for this condition is the use of adrenocorticosteroids and immunosuppressive medications. This type of treatment has shown some efficacy in treating the disease, but recurrence is a possibility, and long-term corticosteroid treatment has serious side effects, such as transient burning or stinging associated with application, local swelling, secondary candidiasis, skin rashes, mucosal atrophy, and dryness [2,3].

As far as OLP is concerned, immune dysregulation plays an important role, and CD8+ cytotoxic lymphocytes and CD4+ Th1-polarized T lymphocytes drive this process, which is triggered by antigens that do not originate from the body, thereby activating T cells that are directed toward oral keratinocytes and causing their death [4]. Darczuk et al. pointed out that free radicals and increased oxidative stress might be involved in the onset and development of an OLP lesion [5]. In addition, according to a recent meta-analysis, OLP shows an imbalance in redox homeostasis that results in increased oxidative stress markers, and antioxidant markers are observably lower in OLP patients than in healthy controls. In summary, the complex pathogenesis of OLP complicates its treatment. A treatment approach based on Chinese medicine has the characteristics of multiple components and multiple targets, thus offering a fresh perspective on the treatment of OLP.

*Tripterygium wilfordii* is a common herbal medicine used to suppress immune function, inhibit fibrosis, inhibit tumor growth, and reduce inflammation in the body [6]. Since it suppresses the immune system and inhibits inflammation, a wide variety of immune disorders benefit from the use of *Tripterygium wilfordii*, for instance, psoriasis, diabetic kidney, and rheumatoid arthritis, which benefit from its pharmacological effects on multiple targets and pathways [7,8,9]. There are some adverse effects associated with *Tripterygium wilfordii*, including liver and kidney toxicity, as well as reproductive toxicity [10]. OLP could be effectively treated with *Tripterygium wilfordii*, as shown in clinical trials [11]. However, the molecular mechanism of *Tripterygium wilfordii*’s action is still unclear. Network pharmacology could guide us in understanding the complex relationships between Chinese herbs and diseases. The network pharmacological approach proposed by Hopkins et al. focuses on the interactions among diseases, drugs, and genes at multiple levels [12]. Molecular docking consists of calculating the affinity of drug molecules for receptor macromolecules, simulating their interaction, and designing new pharmaceuticals utilizing computer simulations [13].

**Figure 1:**
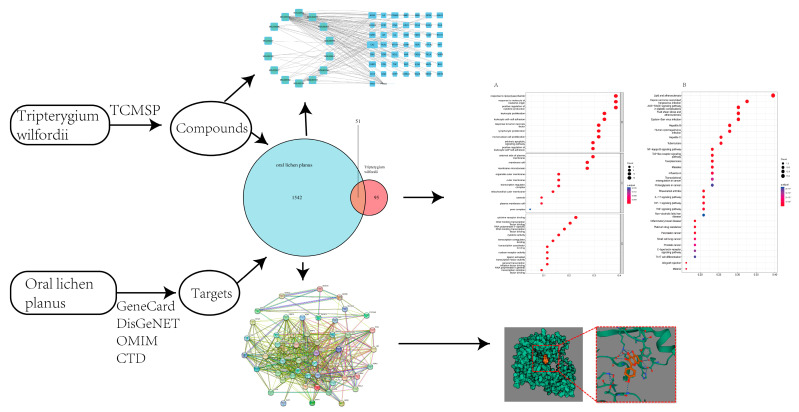
Workflow chart of the pharmacological study on *Tripterygium wilfordii* in the treatment of OLP.

In this study, a network pharmacological analysis was conducted to investigate the mechanism of action of *Tripterygium wilfordii* on OLP. The initial step was to screen *Tripterygium wilfordii* for its active chemical constituents and determine their target proteins. A signaling pathway analysis of gene targets was performed through GO and KEGG. Additionally, cross-validations were conducted on PPI and target proteins. Finally, *Tripterygium wilfordii*’s main target proteins and its active components’ mechanism of binding were determined with the utilization of molecular docking. The detailed procedures can be seen in Figure 1. This article discusses how *Tripterygium wilfordii* may act to treat OLP and provides a reference for future pharmacological laboratory investigations employing molecular docking and network pharmacology.

## 2. Materials and Methods

### 2.1. Screening the Active Components of Tripterygium wilfordii

We utilized the extensive and comprehensive resources of the Traditional Chinese Medicine Systems Pharmacology Database and Analysis Platform (TCMSP) (http://tcmspw.com) (accessed on 11 April 2023), which provided us with valuable insights into this complex process. To ensure high-quality results, we employed an ADME (absorption, distribution, metabolism, and excretion) screening approach, which allowed us to impose stringent selection criteria of drug-likeness (DL) ≥ 0.18 and oral bioavailability (OB) ≥ 30% to efficiently filter out unsatisfactory active ingredients.

### 2.2. Screening the Targets of Active Compounds

HERB (http://herb.ac.cn) (accessed on 13 April 2023) is a high-throughput experiment- and reference-guided database of traditional Chinese medicine, to which the identified active compounds were subjected to obtain their potential targets. In addition, DrugBank (https://www.drugbank.ca/) (accessed on 13 April 2023) was used to identify potential candidate targets. Finally, all target proteins identified during the screening process were annotated with gene names using the UniProt database (https://www.uniprot.org/) (accessed on 13 April 2023), excluding any nonhuman targets.

### 2.3. Gene Screening of OLP-Related Genes

In the pursuit of discovering potential therapeutic targets for “Oral lichen planus” (OLP), sophisticated retrieval systems were employed to scour the vast expanse of relevant data available. We utilized a multi-database approach and turned to the GeneCard database (https://www.genecards.org) (accessed on 14 April 2023), the Online Mendelian Inheritance in Man (OMIM) database (https://omim.org) (accessed on 14 April 2023), the DisGeNET database (https://www.disgenet.org) (accessed on 14 April 2023), and the CTD database (https://www.ctdbase.org) (accessed on 14 April 2023). We generated a comprehensive list of OLP genes from these four disease databases and removed any duplicative entries.

### 2.4. Construction of a Protein–Protein Interaction (PPI) Network

Utilizing the R package “VennDiagram”, we computed the intersection of the detected *Tripterygium wilfordii* targets and related OLP targets, and then a Venn diagram was drawn. Next, capitalizing on the potential of the STRING database (https://string-db.org) (accessed on 16 April 2023), we aggregated the intersection targets and deployed them to construct a PPI network. We set the species type to “Homo sapiens”, ensured the minimum interaction threshold was set to the “highest confidence” (>0.9), and the remaining values were set as the default values. In the end, the results we obtained were imported into the Cytoscape (version 3.9.1) software, which enabled us to construct a PPI network with multiple components, thus providing novel insights into the intricate mechanisms underlying the detected *Tripterygium wilfordii* targets and related OLP targets.

### 2.5. Construction of an Active Compound–Target Network

We harnessed Cytoscape (version 3.9.1) to generate a network to depict the complicated relationships between potential active components and the corresponding OLP targets of *Tripterygium wilfordii*. The nodes in the network represent the genes, activated components, or targets, and the lines indicate the interactions that exist between them. In addition, we picked the top four active components that correspond to the most important potential therapeutic targets of OLP for molecular docking.

### 2.6. Hub Gene Analysis

To extract the hub genes of the PPI network in connection with the *Tripterygium wilfordii* and OLP nexus, the CytoNCA algorithm was employed in CytoScape [14]. This algorithm could calculate the parameters of each node in a network diagram, such as degree, betweenness centrality (BC), closeness centrality (CC), and LAC. We selected the target nodes with degree, BC, CC, and LAC values that were higher than the corresponding median values in the PPI network and predicted the probable core targets of 19 hub genes for further study.

### 2.7. Go and KEGG Enrichment Analyses

To elucidate the effects of 51 target proteins interacting with the target genes of *Tripterygium wilfordii* on gene and gene functions in signaling pathways, we performed GO and KEGG enrichment analyses of potential targets of *Tripterygium wilfordii* intervention in OLP via the R software (version 4.1.2 for Windows). Three aspects were included in the GO enrichment analysis: biological process (BP), molecular function (MF), and cell component (CC). All the GO and KEGG enrichment analysis results were selected based on *p*-values of ≤0.05.

### 2.8. Molecular Docking

The core proteins with the greatest node degree values in the PPI network were docked to their active components. To acquire a complete understanding of the molecular interactions between the compounds and core proteins, we utilized molecular docking techniques. The structure of the target proteins was obtained from the PDB database (http://www.rcsb.org/) (accessed on 18 April 2023), while the drug compounds’ MOL2 structures were obtained from the TCMSP and PubChem (https://pubchem.ncbi.nlm.nih.gov/) (accessed on 18 April 2023) databases. Through the use of PyMOL, we removed water molecules and original ligands from the target proteins and drug compounds. We then utilized the AutoDock Tools (version 1.5.6) to generate charge calculation, non-polar hydrogen combination, and hydrogenation and stored these results in the PDBQT format. Finally, Autodock Vina (version 1.1.2) was used for molecular docking and calculation of minimal binding affinity. According to previous research studies and methodologies, the binding activity of a small-molecule drug to a protein is considered satisfactory when the binding energy is less than −4.25 kcal/mol. Moreover, the binding activity between two molecules is considered excellent when the binding energy is less than −5.0 kcal/mol [15].

## 3. Results

### 3.1. Acquisition of Active Compounds and Targets of Tripterygium wilfordii and Therapeutic Targets for OLP

A total of 51 active compounds of *Tripterygium wilfordii* were obtained by searching the TCMSP database and using the ADME parameters (Table 1). Additionally, 146 *Tripterygium wilfordii* targets were screened from the TCMSP database after deleting duplicated items, and 1593 OLP-related therapeutic targets were screened from the abovementioned four databases.

### 3.2. Venn Diagram

**Figure 2:**
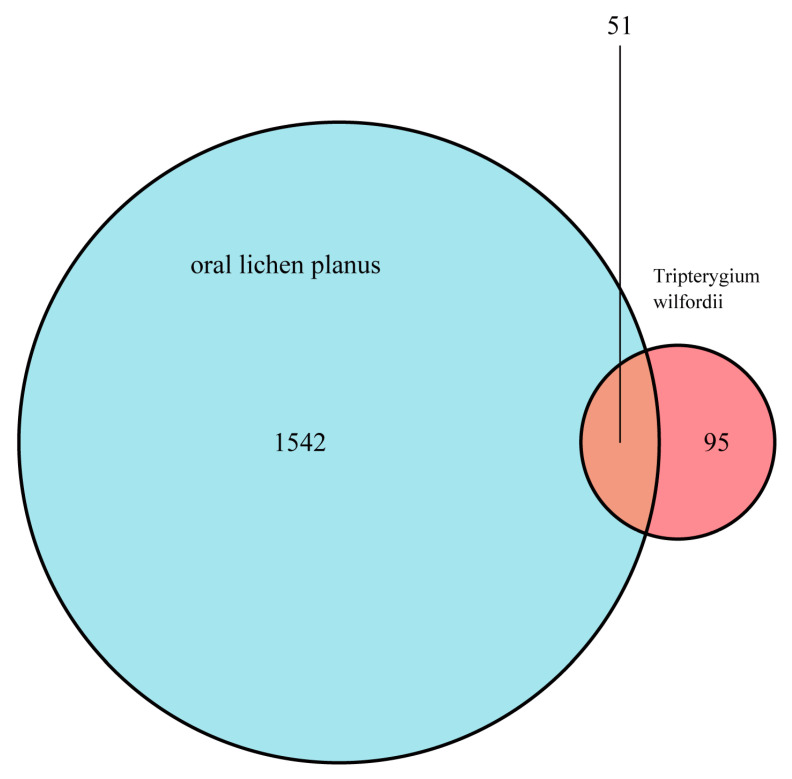
Cross-genes of *Tripterygium wilfordii* effective components and OLP targets.

Our team managed to pinpoint 51 genes of intersection between the targets of *Tripterygium wilfordii* and the disease targets associated with OLP (Table 2). To visualize the results, we used the R software (version 4.1.2 for Windows), which was instrumental in constructing a Venn diagram showcasing the relationship between these two systems (Figure 2).

### 3.3. GO Enrichment and KEGG Pathway Analyses

**Figure 3:**
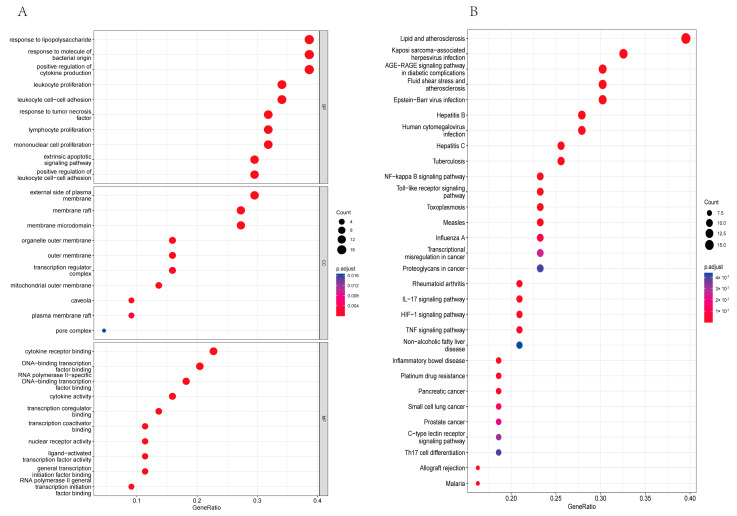
(**A**) The top ten biological processes (BPs), cell components (CCs), and molecular functions (MFs) of Gene Ontology (GO) enrichment analysis are listed in order, from top to bottom. (**B**) The common targets were analyzed based on Kyoto Encyclopedia of Genes and Genomes (KEGG) pathway enrichment. The importance of the top 30 pathways was evaluated and ranked using a bubble diagram.

**Figure 4:**
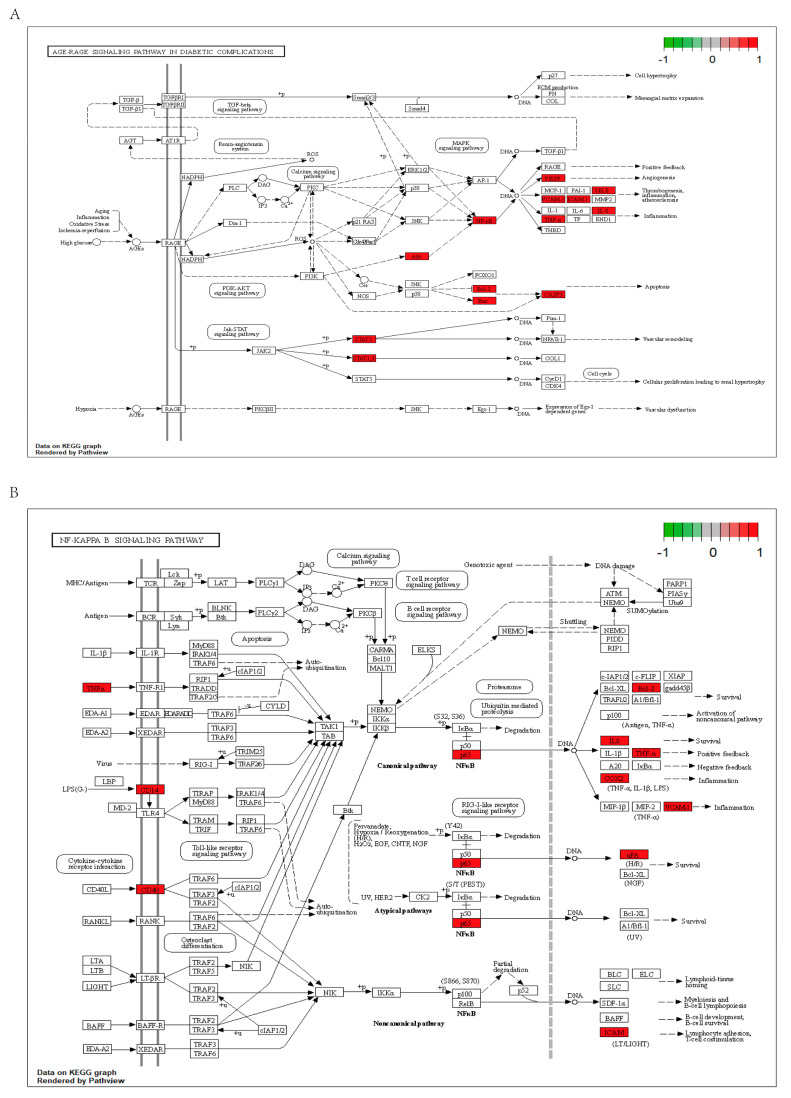
(**A**) Distribution of the target proteins of *Tripterygium wilfordii* on the AGE-RAGE pathways. (**B**) Distribution of the target proteins of *Tripterygium wilfordii* on the NF-κB pathways. The red nodes are potential target proteins of *Tripterygium wilfordii*, while the white nodes are relevant targets in the pathways.

In an effort to uncover the workings of *Tripterygium wilfordii*’s therapeutic capabilities in treating OLP, our team used R software (version 4.1.2 for Windows) to perform GO and KEGG pathway analyses on the 51 common targets. The resulting visual data shown in Figure 3A reveal the top 10 notably enriched GO terms in CC, BP, and MF. To visually describe the complexity of this dataset, we used bubbles of varying sizes, each indicating the number of enriched targets within the relevant pathways. The color coding revealed the −log10(*p*-value), with brighter shades indicating higher levels of enrichment and larger *p*-values being represented by more intense shades of red. The enriched BPs (biological processes) were staggering and spanned the gamut from responses to lipopolysaccharide to positive regulation of cytokine production, and even leukocyte proliferation. The CCs (cellular components) were equally diverse, ranging from the external side of plasma membranes to membrane rafts, membrane microdomains, and organelle outer membranes. The MFs (molecular functions) were just as impressive, with close associations to DNA-binding transcription factor binding, cytokine receptor binding, and cytokine activity. And the KEGG analysis revealed the top 30 biological pathways, including those involved in lipid metabolism and atherosclerosis, Kaposi’s sarcoma-associated herpesvirus infection, AGE-RAGE signaling pathway (Figure 4A), NF-κB signaling pathway (Figure 4B), IL-17 signaling pathway, Toll-like receptor signaling pathway, TNF signaling pathway, and HIF-1 signaling pathway (see Figure 3B).

### 3.4. Diagram of Active Component–Target Network

**Figure 5:**
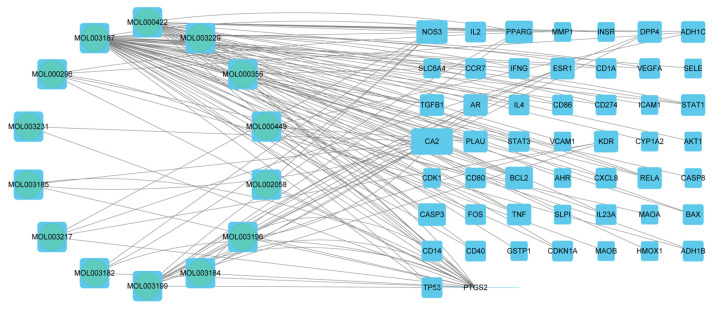
The diagram of the active component–target network. The circles on the left represent the active components of *Tripterygium wilfordii*, and the squares on the right represent the corresponding targets of the active components.

Through Cytoscape, we constructed a *Tripterygium wilfordii* component target network. The resulting figure (Figure 5) reveals the interplay between the many components and targets found within TCM compound prescriptions. The blue squares on the right represent the potential targets of *Tripterygium wilfordii*. The blue squares on the left show the active ingredients of *Tripterygium wilfordii*. Compounds MOL000422 (kaempferol), MOL000358 (β-sitosterol), MOL003187 (triptolide), and MOL000296 (hederagenin) have more targets, thus showing the higher rank these components have in pharmacological action.

### 3.5. PPI Network Construction and Screening of Hub Genes

**Figure 6:**
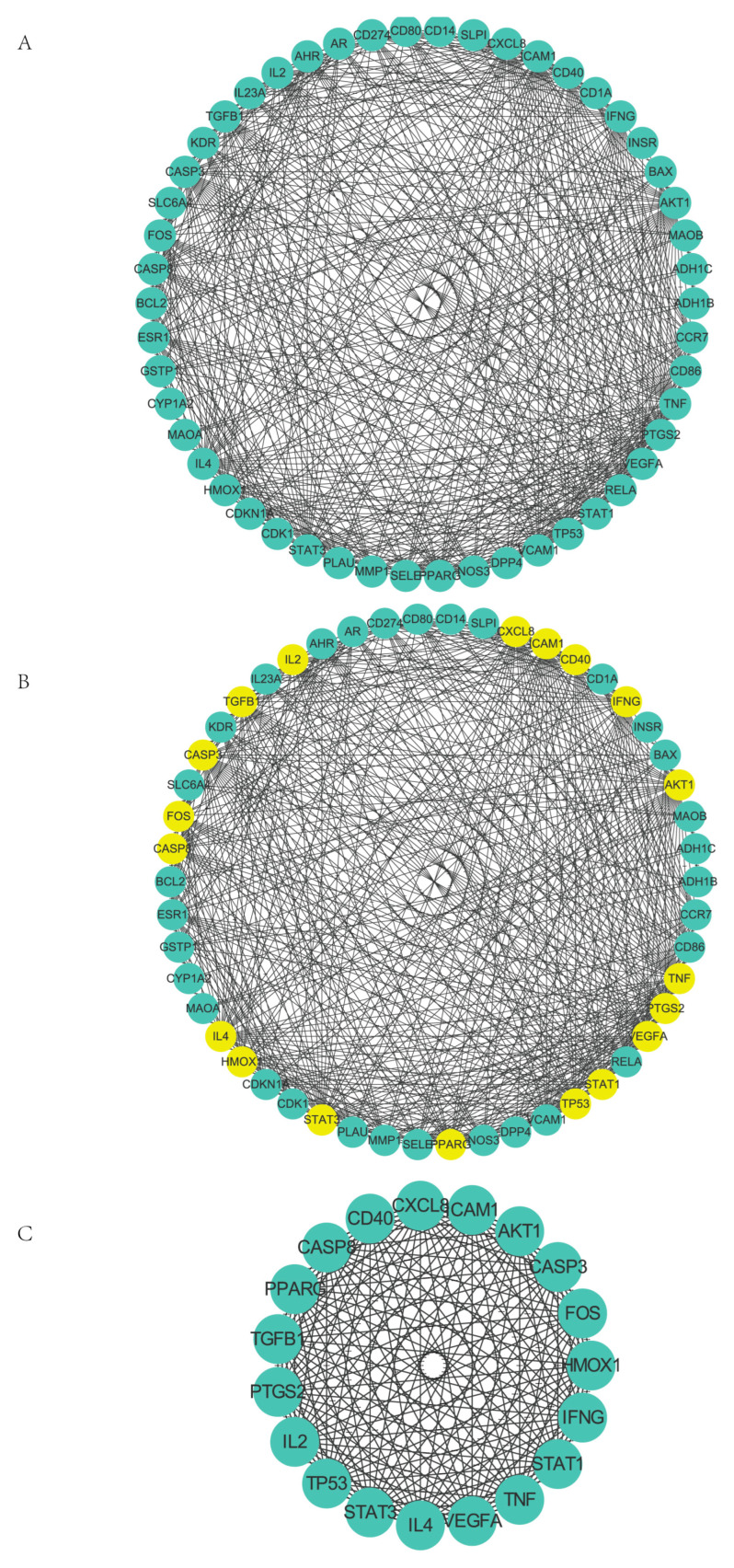
The PPI network and the key genes of the *Tripterygium wilfordii*–OLP common targets. (**A**) PPI network of candidate targets of *Tripterygium wilfordii* against OLP. (**B**) The central top 19 targets are marked by the yellow color. (**C**) The map of the central top 19 targets’ network.

We took our research to the next level by submitting the intersection target proteins to STRING version 11.0, which helped us construct a PPI network consisting of 51 nodes and an astonishing number of 523 edges (Figure 6A). By utilizing CytoNCA, the plug-in of Cytoscape, potential hub genes were screened in the interaction network. We identified the top 19 hub genes of the target proteins, and a hub gene network diagram was constructed (Figure 6B,C). In addition, we picked the top four target proteins (AKT1, CASP3, PTGS2, and TNF) for molecular docking.

### 3.6. Molecular Docking Study

**Figure 7:**
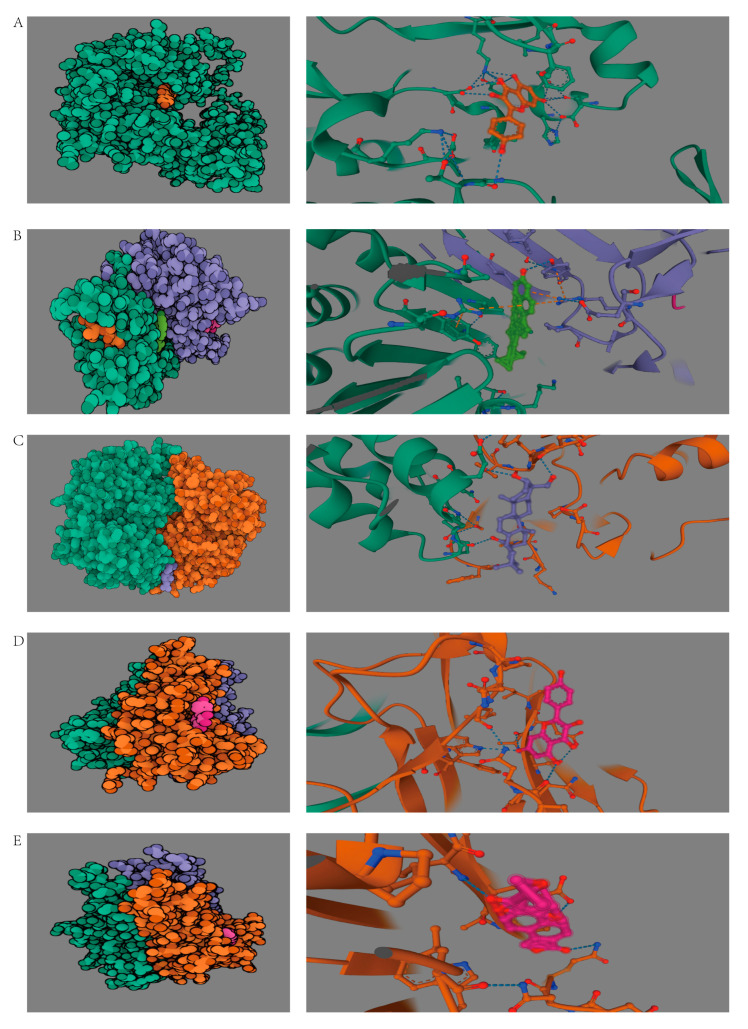
Three-dimensional docking conformations of the targets and the *Tripterygium wilfordii* components with top 5 binding energy values: (**A**) AKT1 and kaempferol, (**B**) CASP3 and β-sitosterol, (**C**) PTGS2 and hederagenin, (**D**) TNF and kaempferol, and (**E**) TNF and triptolide.

We performed molecular docking between the top four compounds (kaempferol, β-sitosterol, triptolide, and hederagenin) and the top four proteins (AKT1, TNF, CASP3, and PTGS2). The results of binding energy are shown in Table 3. Figure 7A shows a representation of the ideal docking of AKT1 to kaempferol, Figure 7B shows the ideal docking of CASP3 to β-sitosterol, Figure 7C shows the ideal docking of PTGS2 to hederagenin, Figure 7D shows the ideal docking of TNF to kaempferol, and Figure 7E shows the ideal docking of TNF to triptolide.

## 4. Discussion

Despite decades of research, the pathophysiological underpinnings of OLP remain shrouded in mystery. Several studies have suggested that immunological and psychological variables may play a significant role in the development and progression of this complex disorder [16]. Furthermore, while conventional treatments for OLP have shown efficacy in many cases, the recurrence rate of this disorder remains stubbornly high, and the side effects associated with long-term hormonal therapy pose a significant impact on the quality of life of affected patients [17]. Against this challenging backdrop, an increasing number of research studies have emerged that highlight the powerful therapeutic potential of TCM in delaying the progression of OLP and strengthen the theory of traditional medicinal approaches to this complex disorder [18,19]. Clinical studies have suggested that *Tripterygium wilfordii* may indeed be a highly promising and safe therapeutic intervention for individuals suffering from OLP [11]. Nevertheless, the multifaceted mechanism underlying its efficacy has remained frustratingly obscure and needs to be resolved.

The innovative methodology of network pharmacology is a revolutionary approach to comprehending the complexity of systems biology and network theory [20]. Network pharmacology has recently been utilized to study the interconnected pathway known as “compound-proteins/genes-disease,” providing insights into the dynamics that define the complex interrelationships that characterize diseases, drugs, and biological systems in a network-centric manner. In light of these remarkable developments, the network pharmacological research method has swiftly become a critical tool in our arsenal for predicting the interrelationship networks that exist between diseases and drugs, thereby facilitating the discovery of novel drugs [21], shedding light on the pharmacological mechanisms that underlie various diseases [22], and uncovering new targets [23].

In this research, we harnessed the power of network pharmacology to unravel the interplay between the major constituents, pivotal pathways, and potential therapeutic targets that underlie *Tripterygium wilfordii*’s efficacy in treating OLP. To bolster the reliability of our findings and conclusions regarding the identified therapeutic targets, we integrated molecular docking into our analytical approach. We found that the main active components of *Tripterygium wilfordii* (kaempferol, β-sitosterol, triptolide, and hederagenin) play vital roles in the treatment of OLP. Kaempferol, the main ingredient of *Tripterygium wilfordii*, has been suggested to protect the vascular endothelial function by reducing oxidative stress and inflammation [24]. Manifesting a remarkable diversity of potent therapeutic properties, β-sitosterol, also commonly known by its epithet “Key to Life,” has been discovered in numerous plants and exhibits robust anti-tumor, anti-inflammatory, antioxidant, and anti-diabetic effects [25]. In a study conducted by Yin Yongxia et al., β-sitosterol was demonstrated to significantly reduce the levels of highly toxic pro-inflammatory mediators in mice, including but not limited to interleukin 6 (IL-6) and tumor necrosis factor (TNF-α). Impressively, this powerful plant-derived compound also significantly boosted the antioxidative activities of essential enzymes like glutathione (GSH) and catalase (CAT), underlining its remarkable significance and versatility as a therapeutic agent [26]. The compound Triptolide is derived from *Tripterygium wilfordii* and is widely employed in the treatment of a myriad of autoimmune and inflammatory diseases, such as rheumatoid arthritis, psoriasis, systemic lupus erythematosus, and nephritis [27]. Researchers have discovered that Triptolide can inhibit the secretion of a wide array of cytokines, adhesion molecules, and chemokines [28]. Furthermore, hederagenin, a potent and highly effective compound, has been shown to display remarkable anti-inflammatory effects and can even improve fibrosis by inhibiting the critical JAK/STAT signaling pathway [29], thereby playing a powerful role as a critical agent in the fight against inflammation and immune dysfunction. Also, hederagenin has been found to display remarkable antioxidative and antiapoptotic effects via the modulation of the Keap1-Nrf2/HO-1/ROS/Bax/Bcl-2 axis [30].

AKT1, TNF, VEGFA, STAT3, CXCL8, PTGS2, TP53, and CASP3 may be significant targets of *Tripterygium wilfordii* in the treatment of OLP, according to the analyses using STRING and CytoNCA. AKT1 is a member of the AKT family and participates in the PI3K/AKT signaling pathway. By responding to extracellular signals, the PI3K/AKT pathway is a necessary regulator of critical cellular functions, such as cell proliferation, growth, angiogenesis, and metabolic processes [31]. This pathway’s critical regulators, including p-Akt and p-mTOR, were found to be significantly elevated in both OLP lesions and local T cells in a ground-breaking study conducted by Zhang et al. This suggests that activated Akt/mTOR autophagy may be a key factor in the local T cell-mediated immune regulatory mechanism of OLP [32]. Additionally, a wealth of evidence has emerged to support the contention that the blood and saliva of OLP patients display markedly increased levels of TNF when compared to healthy controls [33,34,35], underscoring the potential clinical significance of this key cytokine in driving the immunopathogenesis of OLP. Intriguingly, the STAT3 molecule, a crucial member of the STAT family of proteins, has proven to be sensitively activated by upstream cytokines such as TNF-α [36], thereby playing a crucial part in controlling a variety of biological processes, including cell growth and angiogenesis, as well as differentiation and survival. Highlighting the critical importance of the TP53 tumor suppressor gene in controlling key pathways involved in cell cycle regulation and apoptosis, numerous studies have revealed a strong link between TP53 overexpression in OLP and the potential for malignant transformation [37]. Moreover, the activation of the Caspase-3 enzyme, a well-known apoptosis marker, has been shown to modulate gene expression involved in vasculogenesis by favorably down-regulating genes engaged in apoptosis and promoting an increase in vasculogenesis [38].

Based on the GO analysis results, the potential mechanism of *Tripterygium wilfordii* in the treatment of OLP may be related to BPs involved in leukocyte proliferation, positive regulation of the production of cytokines, response to bacterial-derived molecules, and response to lipopolysaccharide. These BP signaling pathways are related to immune response and inflammation. Our investigation suggests *Tripterygium wilfordii*’s capabilities in controlling key cellular processes, such as the regulation of cell growth, apoptosis, and immune response, all of which are of great significance in the etiology and pathogenesis of OLP. A series of intensive research studies have uncovered a myriad of connections between the pathophysiology of OLP and the intricate workings of aberrant T cell activation, the delicate balance of oral keratin-forming cell death, and subtle shifts in the body’s redox state [4,39]. The KEGG enrichment analysis showed that the pharmacological effects of *Tripterygium wilfordii* on OLP are closely related to well-known OLP-associated pathways, such as the NF-κB signaling pathway, the AGE-RAGE signaling pathway, the IL-17 signaling pathway, the Toll-like receptor signaling pathway, the TNF signaling pathway, and the HIF−1 signaling pathway. It has been reported that the Toll-like receptor signaling pathway and the NF-κB signaling pathway may interact in the perpetuation of OLP [40]. Toll-like receptors, being a classic exemplar of pattern recognition receptors, are participants in the complex and subtle mechanisms underlying a vast array of auto-immune disorders. By transducing signals through the MAP kinase and NF-κB signaling pathways, Toll-like receptors unleash a cascade of proinflammatory cytokines and costimulatory molecules that induce inflammatory responses [41]. Several investigations have recently uncovered functions of the IL-17 pathway in a regulated immune network that characterizes the inflammatory environment of OLP lesions. By stimulating keratinocytes to produce a diverse array of inflammatory mediators, the IL-17 signaling pathway unleashes a cascade of inflammatory molecules that lay the groundwork for a host of complex physiological and pathological processes in OLP [42]. According to the obtained data, the network pharmacological analysis revealed that *Tripterygium wilfordii* could hinder OLP growth via various targets and signaling pathways. Furthermore, *Tripterygium wilfordii*’s probable methods of treating OLP include actions on several targets and interactions between the targets. Drugs with multi-target regulatory activity may be more appropriate for diseases with a complicated etiology such as OLP, opening up new avenues for treatment.

To investigate *Tripterygium wilfordii*’s potential molecular mechanism for treating OLP, we screened four vital biologically active ingredients and four representational targets. The four active compounds, namely kaempferol, β-sitosterol, triptolide, and hederagenin, were molecularly docked with a series of vital proteins, including AKT1, TNF, CASP3, and PTGS2, respectively, to validate our network pharmacological predictions. Indeed, with the binding energies below the threshold of −5 kcal/mol, the affinity between these biological molecules is powerful.

Despite the significant findings of our investigation, we must acknowledge that there are certain limitations to our study. While our use of molecular docking and network pharmacological methodologies allowed us to identify several key components and target proteins of *Tripterygium wilfordii* in its treatment of OLP, we recognize that further experiments are required to verify these findings. Additionally, we recognize that current network information technology is by no means perfect, and the databases that we used, while comprehensive, are not without minor flaws, as they occasionally yield false positive results due to limited data availability.

## 5. Conclusions

In the present study, by means of network pharmacology and molecular docking, we conducted a preliminary investigation on the active compounds and mechanism of *Tripterygium wilfordii* in the treatment of OLP. Our results reveal that kaempferol, β-sitosterol, triptolide, and hederagenin are the primary active compounds of *Tripterygium wilfordii* in the treatment of OLP. And AKT1, TNF, CASP3, and PTGS2 are the potential therapeutic targets of *Tripterygium wilfordii* in the treatment of OLP. The above compounds interfere with these targets through various signaling pathways, such as the AGE-RAGE, NF-κB, Toll-like receptor, IL-17, HIF-1, and TNF signaling pathways. Our research provides positive aspects and new horizons for future experimental validation of *Tripterygium wilfordii* in the treatment of OLP and development of Chinese patent medicine.

## Figures and Tables

**Table 1:** Active compounds of *Tripterygium wilfordii*.

Mol ID	Molecule Name	MW	Hdon	Hacc	OB	DL
MOL000296	hederagenin	414.79	1	1	36.91	0.75
MOL003182	(+)-Medioresinol di-O-beta-D-glucopyranoside_qt	388.45	2	7	60.69	0.62
MOL003184	81827-74-9	342.47	1	4	45.42	0.53
MOL003185	(1R,4aR,10aS)-5-hydroxy-1-(hydroxymethyl)-7-isopropyl-8-methoxy-1,4a-dimethyl-4,9,10,10a-tetrahydro-3H-phenanthren-2-one	346.51	2	4	48.84	0.38
MOL003187	triptolide	360.44	1	6	51.29	0.68
MOL003188	Tripchlorolide	396.9	2	6	78.72	0.72
MOL003189	WILFORLIDE A	486.81	2	4	35.66	0.72
MOL003192	Triptonide	344.39	0	6	67.66	0.7
MOL003196	Tryptophenolide	312.44	1	3	48.5	0.44
MOL003198	5 alpha-Benzoyl-4 alpha-hydroxy-1 beta,8 alpha-dinicotinoyl-dihydro-agarofuran	600.72	1	10	35.26	0.72
MOL003199	5,8-Dihydroxy-7-(4-hydroxy-5-methyl-coumarin-3)-coumarin	352.31	3	7	61.85	0.54
MOL003206	Canin	278.33	1	5	77.41	0.33
MOL003208	Celafurine	369.51	2	6	72.94	0.44
MOL003209	Celallocinnine	405.59	2	5	83.47	0.59
MOL003210	Celapanine	569.66	0	11	30.18	0.82
MOL003242	Triptofordinine A2	741.85	1	13	30.78	0.47
MOL003241	Triptofordin F4	652.75	3	12	31.37	0.67
MOL003239	Triptofordin F2	668.75	2	13	33.62	0.67
MOL003238	Triptofordin F1	694.79	2	13	33.91	0.6
MOL003236	Triptofordin D2	650.78	1	11	30.38	0.69
MOL003235	Triptofordin D1	606.72	1	10	32	0.75
MOL003234	Triptofordin C2	610.71	2	11	30.16	0.76
MOL003233	Triptofordin B2	608.69	1	11	107.71	0.76
MOL003232	Triptofordin B1	478.63	1	6	39.55	0.84
MOL003231	Triptoditerpenic acid B	328.49	1	3	40.02	0.36
MOL003229	Triptinin B	314.46	2	3	34.73	0.32
MOL003225	Hypodiolide A	318.5	1	3	76.13	0.49
MOL003224	Tripdiotolnide	360.44	2	6	56.4	0.67
MOL003222	Salazinic acid	402.33	4	10	36.34	0.76
MOL003217	Isoxanthohumol	354.43	2	5	56.81	0.39
MOL003211	Celaxanthin	550.94	1	1	47.37	0.58
MOL003210	Celapanine	569.66	0	11	30.18	0.82
MOL003209	Celallocinnine	405.59	2	5	83.47	0.59
MOL003208	Celafurine	369.51	2	6	72.94	0.44
MOL003206	Canin	278.33	1	5	77.41	0.33
MOL003199	5,8-Dihydroxy-7-(4-hydroxy-5-methyl-coumarin-3)-coumarin	352.31	3	7	61.85	0.54
MOL003198	5 alpha-Benzoyl-4 alpha-hydroxy-1 beta,8 alpha-dinicotinoyl-dihydro-agarofuran	600.72	1	10	35.26	0.72
MOL003196	Tryptophenolide	312.44	1	3	48.5	0.44
MOL003192	Triptonide	344.39	0	6	67.66	0.7
MOL003189	WILFORLIDE A	486.81	2	4	35.66	0.72
MOL003188	Tripchlorolide	396.9	2	6	78.72	0.72
MOL003187	triptolide	360.44	1	6	51.29	0.68
MOL003185	(1R,4aR,10aS)-5-hydroxy-1-(hydroxymethyl)-7-isopropyl-8-methoxy-1,4a-dimethyl-4,9,10,10a-tetrahydro-3H-phenanthren-2-one	346.51	2	4	48.84	0.38
MOL003184	81827-74-9	342.47	1	4	45.42	0.53
MOL003182	(+)-Medioresinol di-O-beta-D-glucopyranoside_qt	388.45	2	7	60.69	0.62
MOL002058	40957-99-1	388.45	2	7	57.2	0.62
MOL000449	Stigmasterol	412.77	1	1	43.83	0.76
MOL000422	kaempferol	286.25	4	6	41.88	0.24
MOL000358	beta-sitosterol	414.79	1	1	36.91	0.75
MOL000296	hederagenin	414.79	1	1	36.91	0.75
MOL000211	Mairin	456.78	2	3	55.38	0.78

Note: MW: molecular weight; Hdon: hydrogen bond donors; Hacc: hydrogen bond acceptors; OB: oral bioavailability; DL: drug-likeness.

**Table 2:** Intersection genes between OLP and *Tripterygium wilfordii*.

Intersection Gene Name									
ADH1B	RELA	CDKN1A	STAT1	IL4	CCR7	AR	MAOA	HMOX1	GSTP1
ADH1C	STAT3	PLAU	CXCL8	CD80	CD1A	PPARG	AKT1	CYP1A2	AHR
PTGS2	VEGFA	TNF	TGFB1	CD86	CD40	KDR	BAX	ICAM1	INSR
NOS3	BCL2	CASP3	IL2	CD274	CD14	DPP4	MMP1	SELE	SLPI
CA2	FOS	TP53	IFNG	IL23A	ESR1	MAOB	CDK1	VCAM1	SLC6A4
CASP8									

**Table 3:** Docking parameters and results.

No.	Target	PDB ID	Compound	Minimum Binding Energy (kcal/mol)
1	AKT1	5AAR	kaempferol	−8.592
2	CASP3	5JFT	β-sitosterol	−11.912
3	PTGS2	1PXX	hederagenin	−8.175
4	TNF	4QPY	kaempferol	−7.266
5	TNF	4QPY	triptolide	−8.385

## Data Availability

The data presented in this study are available from the corresponding author upon request.

## Abbreviations

**OLP**	Oral lichen planus
**KEGG**	Kyoto Encyclopedia of Genes and Genomes
**GO**	Gene Ontology
**PPI**	Protein–protein interaction
**WHO**	World Health Organization
**TCMSP**	Traditional Chinese Medicine Systems Pharmacology Database and Analysis Platform
**ADME**	Absorption, distribution, metabolism, and excretion
**DL**	Drug-likeness
**OB**	Oral bioavailability
**OMIM**	Online Mendelian Inheritance in Man
**CTD**	Comparative Toxicogenomics Database
**AKT1**	AKT serine/threonine kinase1
**TNF**	Tumor necrosis factor
**VEGFA**	Vascular endothelial growth factor A
**TP53**	Tumor protein53
**STAT3**	Signal transduction and transcriptional activator 3
**CASP3**	Caspase 3
**CXCL8**	C-X-C motif chemokine ligand 8
**PTGS2**	Prostaglandin-endoperoxide synthase 2
**BP**	Biological process
**CC**	Cellular component
**MF**	Molecular function
**TCM**	Traditional Chinese medicine
